# Case Report: Challenges in the surgical treatment of Marfan-associated aortic aneurysms: a literature review starting from a clinical case

**DOI:** 10.3389/fsurg.2025.1715026

**Published:** 2026-01-30

**Authors:** Ombretta Martinelli, Antonio Marzano, Valeria Gonta, Lucio Ferriero, Carola D’Amico, Simone Cuozzo, Maria Irene Bellini

**Affiliations:** 1 Department of Surgery “Paride Stefanini”—Policlinico Umberto I, Vascular Surgery Division, Sapienza University, Rome, Italy; 2 Department of Diagnostic Imaging, Oncological Radiotherapy and Hematology—Fondazione Policlinico Universitario A. Gemelli IRCCS, Rome, Italy; 3 Department of Surgery, Sapienza University, Rome, Italy

**Keywords:** aortic aneurysm, cardiovascular disease, case report, endovascularaneurysm repair, Marfan syndrome, open aneurysm repair

## Abstract

Marfan syndrome (MFS) is a systemic connective tissue disease severely affecting the cardiovascular system. We present the case of a MFS 55-year-old woman who arrived at the emergency department with increasing chest pain. Over the past 25 years, this patient had undergone mitral valve annuloplasty, subsequent open surgical repair of a ruptured infrarenal abdominal aortic aneurysm followed by open surgery for a type I thoracoabdominal aortic aneurysm. She was also operated for fenestrated endovascular repair of a visceral aortic aneurysm using a ‘graft-to-graft’ approach. Upon the urgent admission, a multislice computed tomography angiography demonstrated an aortic aneurysm sac with a maximum diameter of 11.8 cm that was fed by a type IIIB endoleak, due to complete branch stent disconnection of the right renal artery (RRA) and by type IIIB/IIIC endoleaks secondary to stent fracture and disconnection in the superior mesenteric artery (SMA) and celiac trunk (CT), respectively. A common hepatic artery aneurysm (diameter of 2.29 cm) was detected, too. Under general anaesthesia a relining of both RAA and SMA was performed with Ballon-expandable Gore Viabahn and VBX stent-grafts. Subsequently, a CT stenting was successfully carried out. The bridging stents were intentionally positioned to protrude into the fenestrations to get enough overlap with the previously placed stent at the target vessel level. There were no postoperative systemic complications and the patient was discharged after 3 days under dual anti-platelet therapy. At 12 months of follow-up, complete exclusion and shrinkage of the aneurysmal sac and the patency of the stented visceral vessels are demonstrated. This complex case serves as the starting point for a literature review on current trends and perspectives in the treatment of aortic pathology related to MFS. Since MFS patients often present with aortopathy at a young age, different surgical treatments could be combined over the years to provide durable results in in protection against aortic rupture, until more effective drugs can be implemented.

## Introduction

The Marfan syndrome (MFS) is one of the most common single-gene malformation syndrome predominantly due to fibrillin 1 gene (FBN1) mutations leading to the increase of TGF-beta and subsequent activation of matrix metalloproteinases (MMPs), particularly MMP-2 and MMP-9, cytokines, chemokines, prostaglandin derivatives (1), as well as affecting BMP signalling. These alterations cause elastin and collagen destruction which compromise the structural and functional integrity of all connective tissues, predisposing cardiac damages, among which mitral and tricuspid valve prolapse, cardiomyopathy, and dilatation of the aortic root (2). Moreover, there is extensive mitochondrial dysfunction, as a direct consequence of reduced FBN1 extracellular matrix, which exhausts the vascular cells. This connective disease also leads to progressive aortic dilatation and dissections which are important causes of decreased life expectancy, requiring treatment (3, 4).

Yet, the systemic nature of connective tissue weakening and the unpredictable progression of Marfan-associated aortic damages make their management challenging with a high risk of complications.

We report a case of MFS, which is extraordinary for the sequential dilation of different aortic segments in a single patient necessitating an increasingly complex management, not free from complications. We used such a complex case as a reference for literature review on current trends and perspectives in the treatment of aortic pathology related to MFS.

## Case

A 55-year-old woman was admitted to our Emergency Department complaining of chest pain. The patient's medical history included MFS, arterial hypertension, obesity (class II, BMI 37,1 kg/m^2^), and laparocele with a hostile abdomen.

Due to her connective tissue disorder, over the past 25 years, the patient had undergone multiple cardiovascular surgeries carried out in various hospital centres.

The patient underwent mitral valve annuloplasty with a Carpentier-Edwards ring to treat major mitral valve prolapse at the age of 29 years. In 2001 she underwent open surgical repair via median laparotomy for a ruptured infrarenal abdominal aortic aneurysm (AAA) followed by open surgery via thoraco-phreno-laparotomy for a type I thoracoabdominal aortic aneurysm (TAAA) in 2003. In 2015, due to the subsequent development of a suprarenal abdominal aortic aneurysm, she was submitted to a fenestrated endovascular repair (FEVAR) using a ‘graft-to-graft’ approach with a custom-made stent-graft (Cook Medical Inc., Bloomington, IN, USA). At the time of the suprarenal aortic dilatation, endovascular intervention was justified regardless of long-term stability of the repair due to the patient's high operative risk and difficulties of a new open surgical approach.

**Figure 1:**
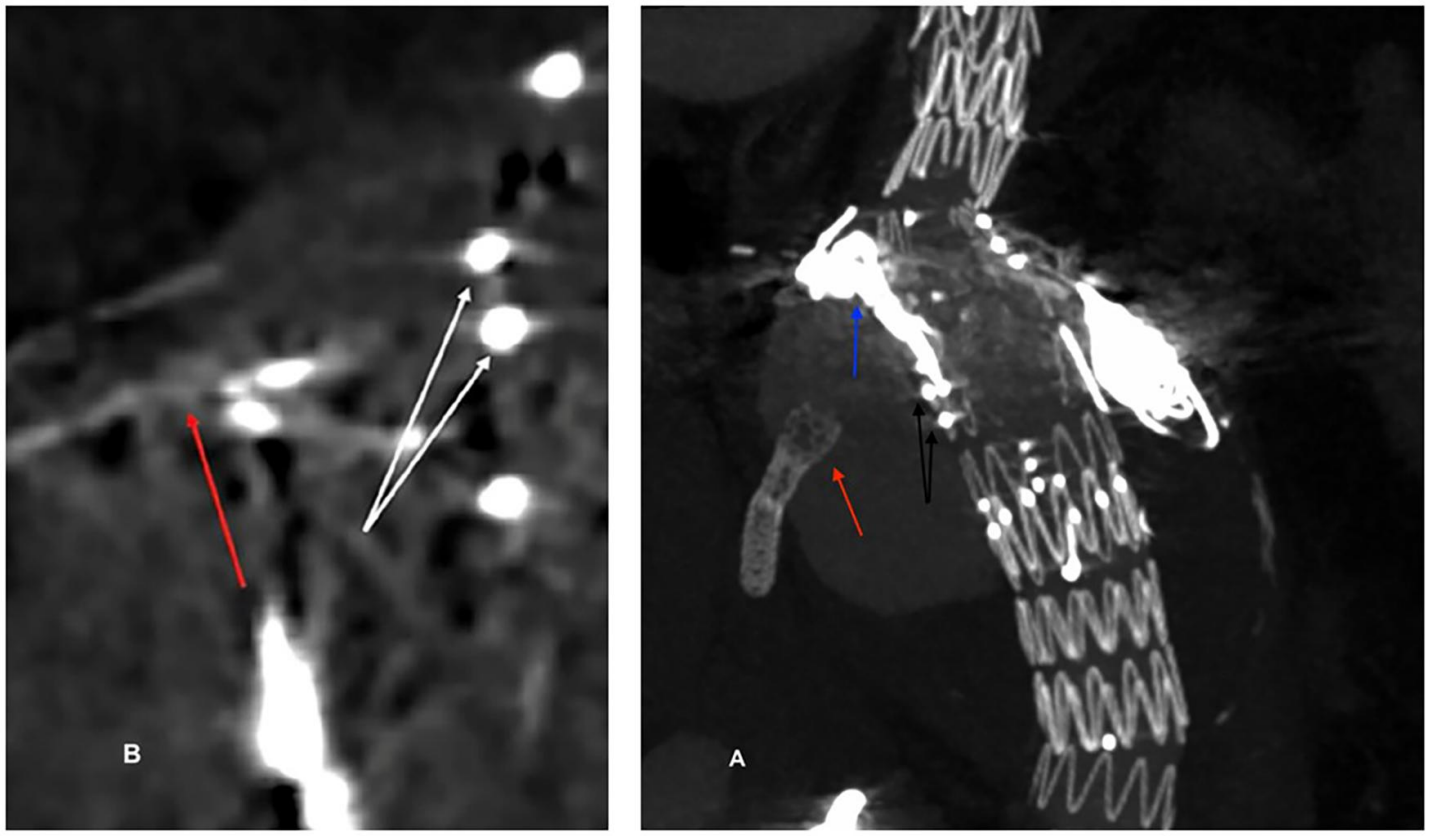
3D reconstruction of the preoperative CT scan. (A) Type IIIB endoleak due to a disconnection between the stent (red arrow) and the fenestration (black arrows) of the RRA. The coils within the aneurysmal sac are also visible (blue arrow). **(B)** Type IIIB/IIIC endoleak due to a disconnection and fracture between the stent (red arrow) and the fenestration (white arrows) of the SMA. RRA, right renal artery; SMA, superior mesenteric artery.

The computed tomography angiography (CTA) carried out on emergency admission (Figure 1) showed a type IIIB endoleak caused by a complete stent disconnection of the right renal artery (RRA) and to a type IIIB/IIIC endoleak due to stent fracture and disconnection of the superior mesenteric artery (SMA); these endoleaks fed the aneurysm sac which had reached a maximum transverse diameter of 11.8 cm. On CTA, the bridging stent in the celiac trunk (CT) was not detected because of its absence or destruction; contemporarily, aneurysm of distal common hepatic artery, with a maximum transverse diameter of 2.29 cm, was also observed.

Under general anaesthesia and through bilateral percutaneous femoral access with a 16 F steerable catheter sheath (Aptus HeliFX, Medtronic, Minneapolis, USA) a relining of the RAA and of the SMA was performed with Ballon-expandable Gore Viabahn and VBX stent-grafts (W.L. Gore & Associates, Flagstaff, AZ, USA). Subsequently, CT stenting was successfully carried out. The bridging stents were intentionally positioned to protrude into the fenestrations, in order to ensure also sufficient overlap with the previously placed stent at the target vessel level.

**Figure 2:**
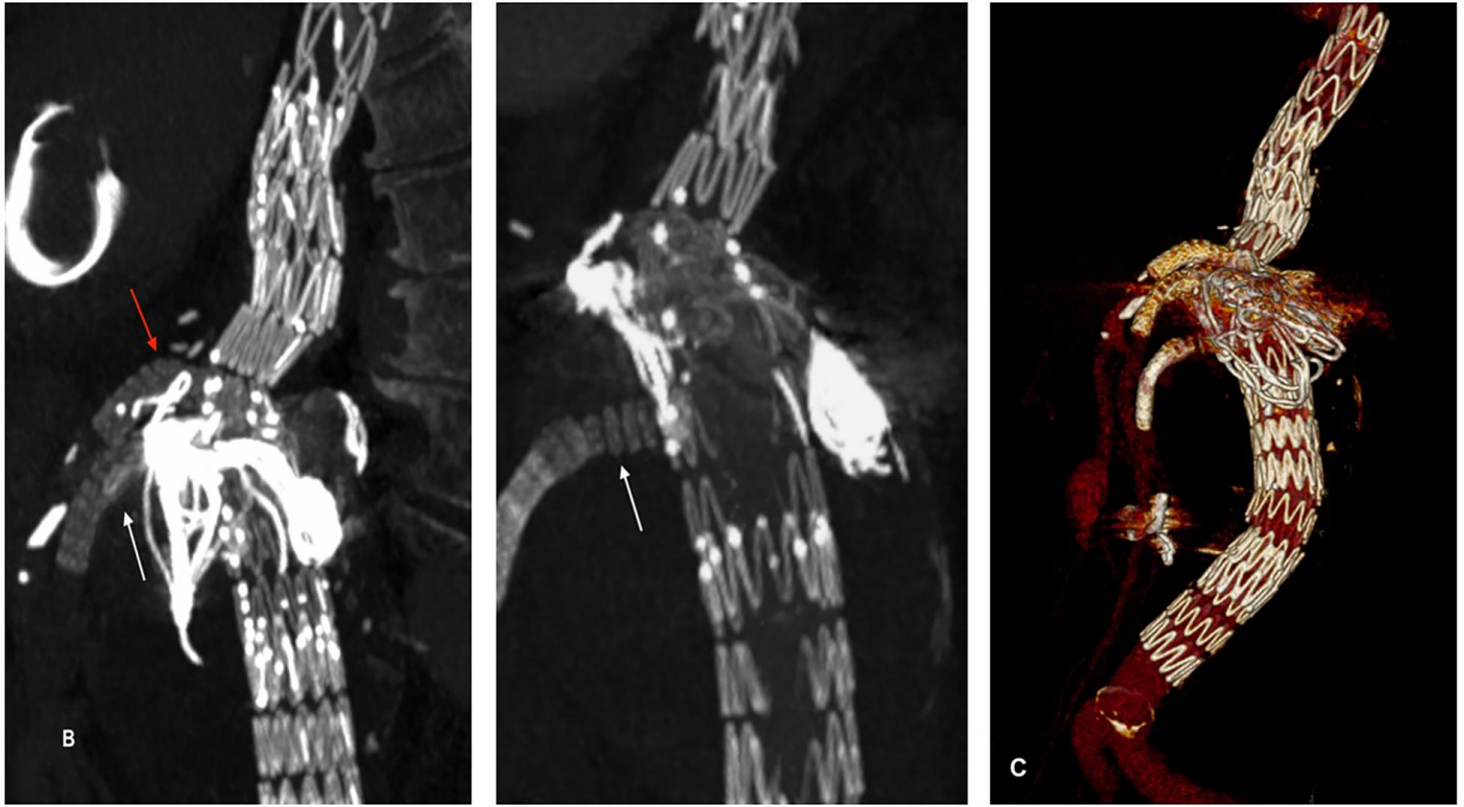
3D reconstructions of the 12-month CTA showing correct positioning and patency of the stents in the RRA, SMA, and CT, with no evidence of endoleaks. **(**A) relining of the RRA stent (white arrow). **(**B) relining of the SMA stent (white arrow) and placement of the stent in the CT (red arrow). **(**C) volume rendering. CT, celiac trunk; SMA, superior mesenteric artery; RRA, right renal artery.

There were no systemic complications during or after the procedure and the patient was discharged after 3 days under dual anti-platelet therapy. Duplex ultrasounds performed at 1, 3, 6 and 12 months and CTA performed in the first month and at 12 months of follow-up demonstrated the complete exclusion and shrinkage of the aneurysmal sac and the patency of the stented visceral and renal vessels (Figure 2). Consent was obtained from the patient for publication of the case report.

## Discussion

MFS is a serious chronic disorder involving multiple organ systems with no cure at present. In patients with MFS, *FBN1* gene mutation leads to a structural weakness of connective tissue due to the elastin and collagen destruction, and predisposes to aortic root aneurysm and aortic aneurysm and dissection (5–7).

Life-threatening heart valve complications requiring replacement often occur at a younger age in MFS patients, compared to other degenerative aortic disease and involving the thoracic aorta, more frequently than abdominal aorta (8, 9).

Since, medical therapy of MFS with beta-blockers and type 1 angiotensin II receptor blockers can slow down aortic aneurysm progression but could not completely arrest aneurysmal growth, aortic surgery is still the only treatment to prevent aneurysmatic rupture (10, 11).

Therefore, MFS patient survival is closely related with the severity of aortic involvement and the timeliness of its diagnosis and treatment (12). Although true isolated abdominal aortic aneurysms (AAA) represent only 3% of initial aortic disease in MFS, this atypical manifestation of MFS must be carefully considered as early diagnosis and timely intervention influence long-term prognosis (13). The case presented herein is emblematic for the early onset and the possible atypical pattern of cardiovascular diseases in MFS patients: the patient required mitral valve surgery at 29 years and emergency open repair of a ruptured infrarenal AAA at the age of 31.

The fragility of the aortic wall and the young age of these patients with the inherent lifelong propensity toward progressive aortic dilation and dissection make repair selection particularly challenging (14). It is well known in fact that MFS patients continue to experience aortic degeneration throughout their lives and often require several sequential aortic surgical procedures (15). Consistently with the exceptional course of the disease, two years after infrarenal AAA replacement surgery, this patient had a type I TAAA and 12 years later she experienced a further progression of her aortic pathology, leading to dilatation of the remnant segment of the descending aorta. Both the ruptured AAA and subsequent type I TAAA were treated with open repair.

Although endovascular repair has become standard for many degenerative aneurysms due to lower early morbidity and mortality (16–18), it remains a controversial option in connective tissue disorders (19, 20). In MFS, open surgical repair remains the recommended treatment for aortic aneurysms, including thoracoabdominal aortic aneurysm, given the possibility to remove diseased tissue, thus providing the most durable long-term protection (21).

Highly satisfactory outcomes with open repair of aortic disease in MFS patients have been reported by several surgical centers (21, 22); in particular, Coselli et al. reported on the excellent survival and low re-operation rates after open surgery in connective tissue disorders, including MFS up to 8 years after thoracoabdominal aortic open repair (23).

A further consideration in favor of open surgery is younger age of MFS patients, in comparison to the general aortic diseases population. These patients usually have low operative risk, but the longest durability of the repair is crucial, given the life-expectancy.

The current propensity for open surgery also derives from the potential impact of persistent radial forces of a stent graft on a weak and progressively dilating aorta with subsequent risk of stent displacement, complications and need for open conversion repair (24).

Notwithstanding, the rapid technology evolution and improvement of the results in the endovascular treatment of aortic pathology have led to a reconsideration of its use for MFS patients (25–27). Multiple reports have described high technical success, low early mortality and good short-term results of aortic endo-grafting in these patients (28, 29).

From a literature review by Harky et al. 81% of the endovascular procedures were technically successful with a 1.9% risk of death, 1.9% risk of stroke, <2% risk of spinal ischaemia, and 3.7% rate of conversion to open surgery (30).

However, long-term data regarding the endovascular approach in MFS patients show markedly higher rates of endoleaks, device failure, reintervention, and late conversion to open repair, in comparison to aortic degenerative disease (15, 31–35) (Table 1).

**Table 1:** Outcomes of endovascular treatment of aortic disease in patients with connective tissue disorders.

Study	Patients (*N*=)	Median follow-up, months	Endograft-related complications %	Reintervention in same aortic segment (Open) %	Reintervention in same aortic segment (Endovascular) %
Waterman et al. (15)	16	9.3	30	33	13
Pacini et al. (29)	50	24	16	14	16
Glebova et al. (30)	216	9.3–162	44	50	36
Preventza et al. (31)	60	28	17	7	12
Olsson et al. (32)	171	56.4	Not specified	39.8	8.2
Conway et al. (33)	29	25.5	13.7	3.4	6.9

Given the highly satisfactory results with open surgical repair reported over the last decade by several experienced surgical centres, at present, there is no justification for elective endovascular stenting in patients with Marfan syndrome and type B dissection (36) or with aneurysm in the absence of dissection. For these reasons, guidelines from the European Society of Cardiology (ESC), European Association for Cardio-thoracic surgery (EACTS), American Heart Association (AHA), and American College of Cardiology (ACC) restrict endovascular repair in MFS population only as bridge to definitive open surgical therapy or for emergency procedures (37–39).

However, it should be considered that MFS patients previously treated with open aortic surgery may experience progression of their aortic pathology over time requiring further treatments. In 20% of cases, in fact they require intervention on non-aortic arterial segments from 5 to 6 years after the first aortic repair. Surgery then becomes particularly challenging in the context of multiple operations (40). An exception to guidelines recommendation is the situation in which an endograft can be anchored both proximally and distally to previously placed synthetic grafts. Several studies have reported successful outcomes after previous open repair of thoracic or thoracoabdominal aortic aneurysms where the stent grafts were anchored proximally and distally into the preexisting graft (41, 42). In this case the risk of stent graft-induced damages to the friable aortic wall can be avoided. In line with this concept in the present case, the fenestrated endograft was deployed between two existing surgical grafts and this ‘graft-to-graft’ approach effectively circumvented the risk of landing zone enlargement and distal migration (43). However, the long-term remodelling of the aorta and visceral vessels led to instability at the bridging stent interfaces, resulting in type III endoleaks. This occurrence, as observed in the present case, required urgent endovascular relining for late type IIIB endoleak and a type IIIC secondary to the disconnection of the bridging stents inserted in the RRA and SMA.

This highlights a crucial vulnerability of fenestrated and branched repair systems: the connection between the main graft and bridging stents is a frequent failure point, even more likely in patients with connective tissue disorders. Beyond progressive aortic dilatation, patients affected by MFS frequently develop aneurysmal degeneration of the visceral and iliac arteries (44, 45).

Reported series describe a high prevalence of branch vessel aneurysms, reaching up to 27%, together with a markedly increased incidence of iliac aneurysmal progression after AAA repair (8, 46).

Such vascular changes heighten the risk of branch-related failure and endoleaks and predispose to extra-aortic aneurysms as exemplified by this case, which was further complicated by a concomitant common hepatic artery aneurysm managed under active surveillance.

Although ongoing advances in endograft design may ultimately mitigate the frequency or possibly eliminate the complications associated with currently available devices, their safety and effectiveness have yet to be conclusively demonstrated (47, 48). In the absence of robust clinical evidence establishing long-term safety and durability, the routine use of endovascular stent grafts in patients with MFS cannot be recommended and should be reserved for highly selected cases. Additionally, it would be desirable to combine surgical or endovascular treatment with effective medical therapies aiming to slow down aortic growth. About this, the current evidence of pharmacological treatment for MFS patients is conflicting due to the lack of large trials (49). Beta-blockers, and angiotensin receptor blockers are the only available treatments to reduce aortic growth (49). Currently, doxycycline, is under consideration but its use is controversial due to the negative effect on mitochondria. These structures are in fact of crucial importance to aneurysm formation in MFS, in fact the distortion of the extracellular matrix-mitochondrial homeostasis axis intensifies aortic wall reorganisation, evolving into aneurysmatic sac (50). On the contrary, nicotinamide riboside, shows promise in aneurysm animal models by boosting mitochondrial function, improving vascular smooth muscle cell health, and reversing aortic dilation (51). The same beneficial effect might be observed with resveratrol, a dietary supplement interfering with cellular metabolism, as it stabilises the aortic growth rate in adult patients with MFS, according to a recent clinical trial (52). Finally, growing interest is also toward allopurinol, normally used against gout, as it is proven to hamper aortic aneurysm progression in MFS mice by its antioxidant mechanisms (53). Thus novel pharmacological therapies seem upcoming.

## Conclusions

The present case highlights several practical frameworks for managing complex or redo aortic pathology in MFS patients: open surgery remains the first-line therapy for most elective aortic aneurysms and the routine use of endovascular stent grafts is not recommended.

However, endovascular aortic repair cannot be considered a prohibitive indication, but it should be reserved for selected cases such as primarily emergencies, high-risk reoperations, or surgically challenging cases as part of a multistage aortic repair with graft-to-graft landing zones following prior open aortic repairs.

Visceral and iliac arteries must be carefully monitored, as their progressive dilation predispose to extra-aortic aneurysms and can compromise endograft stability.

Although current medical therapy remains limited in its capacity to alter long-term disease progression, emerging treatments show preliminary promise to be validated in large clinical trials. Combining improved medical therapy with tailored open or endovascular strategies may ultimately enhance the durability of repairs in the MFS population.

These observations support the necessity for rigorous, lifelong imaging surveillance of disease progression for timely, anatomy-driven reinterventions, in order to ensure durable management.

## Data Availability

The original contributions presented in the study are included in the article/Supplementary Material, further inquiries can be directed to the corresponding author.

## References

[1] Asta L D'Angelo GA Marinelli D Benedetto U. Genetic basis, new diagnostic approaches, and updated therapeutic strategies of the syndromic aortic diseases: Marfan, loeys-dietz, and vascular ehlers-danlos syndrome. Int J Environ Res Public Health. (2023) 20(16). 10.3390/ijerph20166615 37623198 PMC10454608

[2] Takeda N Yagi H Hara H Fujiwara T Fujita D Nawata K Pathophysiology and management of cardiovascular manifestations in Marfan and loeys-dietz syndromes. Int Heart J. (2016) 57(3):271–7. 10.1536/ihj.16-094 27181042

[3] Vanem TT Geiran OR Krohg-Sørensen K Røe C Paus B Rand-Hendriksen S. Survival, causes of death, and cardiovascular events in patients with Marfan syndrome. Mol Genet Genomic Med. (2018) 6(6):1114–23. 10.1002/mgg3.489 30393980 PMC6305663

[4] Groth KA Stochholm K Hove H Andersen NH Gravholt CH. Causes of mortality in the Marfan syndrome(from a nationwide register study). Am J Cardiol. (2018) 122(7):1231–5. 10.1016/j.amjcard.2018.06.034 30149886

[5] Milleron O Arnoult F Delorme G Detaint D Pellenc Q Raffoul R Pathogenic FBN1 genetic variation and aortic dissection in patients with Marfan syndrome. J Am Coll Cardiol. (2020) 75(8):843–53. 10.1016/j.jacc.2019.12.043 32130918

[6] den Hartog AW Franken R Zwinderman AH Timmermans J Scholte AJ van den Berg MP The risk for type B aortic dissection in Marfan syndrome. J Am Coll Cardiol. (2015) 65(3):246–54. 10.1016/j.jacc.2014.10.050 25614422

[7] de Beaufort HWL Trimarchi S Korach A Di Eusanio M Gilon D Montgomery DG Aortic dissection in patients with Marfan syndrome based on the IRAD data. Ann Cardiothorac Surg. (2017) 6(6):633–41. 10.21037/acs.2017.10.03 29270375 PMC5721116

[8] Schoenhoff FS Yildiz M Langhammer B Jungi S Wyss TR Makaloski V The fate of nonaortic arterial segments in Marfan patients. J Thorac Cardiovasc Surg. (2019) 157(6):2150–6. 10.1016/j.jtcvs.2018.10.089 30578062

[9] Taub CC Stoler JM Perez-Sanz T Chu J Isselbacher EM Picard MH Mitral valve prolapse in Marfan syndrome: an old topic revisited. Echocardiography. (2009) 26(4):357–64. 10.1111/j.1540-8175.2008.00825.x 19054044

[10] van Karnebeek CD Naeff MS Mulder BJ Hennekam RC Offringa M. Natural history of cardiovascular manifestations in Marfan syndrome. Arch Dis Child. (2001) 84(2):129–37. 10.1136/adc.84.2.129 11159287 PMC1718664

[11] Takayama T Miyata T Nagawa H. True abdominal aortic aneurysm in Marfan syndrome. J Vasc Surg. (2009) 49(5):1162–5. 10.1016/j.jvs.2008.12.007 19307079

[12] Heuvel LJF VD Peeters S Meester JAN Coucke PJ Loeys BL. An exploration of alternative therapeutic targets for aortic disease in Marfan syndrome. Drug Discov Today. (2024) 29(7):104023. 10.1016/j.drudis.2024.104023 38750929

[13] Saraber PJMH Bica LC Reesink KD Mees BME Bidar E Schurgers LJ The clinical relevance of the decelerating effect of angiotensin receptor blockers on aortic growth in Marfan patients; a Bayesian perspective. Int J Cardiol. (2025) 433:133318. 10.1016/j.ijcard.2025.133318 40288542

[14] Nordon IM Hinchliffe RJ Holt PJ Morgan R Jahangiri M Loftus IM Endovascular management of chronic aortic dissection in patients with Marfan syndrome. J Vasc Surg. (2009) 50(5):987–91. 10.1016/j.jvs.2009.05.056 19632806

[15] Waterman AL Feezor RJ Lee WA Hess PJ Beaver TM Martin TD Endovascular treatment of acute and chronic aortic pathology in patients with Marfan syndrome. J Vasc Surg. (2012) 55(5):1234–40; disucssion 1240-1. 10.1016/j.jvs.2011.11.089 22465552

[16] Sirignano P Piffaretti G Ceruti S Orso M Picozzi M Ricci G Insight from an Italian delphi consensus on EVAR feasibility outside the instruction for use: the SAFE EVAR study. J Cardiovasc Surg (Torino). (2024) 65(3):273–9. 10.23736/S0021-9509.23.12906-5 38319647

[17] Smedile G Bellini MI Iaria G Castrucci T De Luca L Leporelli P Emergency endovascular repair in a patient with abdominal aortic aneurysm with pelvic transplant kidneys: case report. Exp Clin Transplant. (2012) 10(6):601–4. 10.6002/ect.2012.0017 22765312

[18] Martinelli O Cuozzo S Miceli F Gattuso R D'Andrea V Sapienza P Elective endovascular aneurysm repair (EVAR) for the treatment of infrarenal abdominal aortic aneurysms of 5.0–5.5 cm: differences between men and women. J Clin Med. (2023) 12(13):4364. 10.3390/jcm12134364 37445398 PMC10342711

[19] Cuozzo S Sbarigia E Jabbour J Marzano A D'Amico C Brizzi V Impact of frailty on outcomes of patients undergoing elective endovascular thoraco-abdominal aortic aneurysm repair. J Cardiovasc Surg (Torino). (2024) 65(6):515–22. 10.23736/S0021-9509.24.13052-2 39441148

[20] Martinelli O Di Girolamo A Irace L Baratta F Gossetti B Gattuso R. Post-implantation syndrome: the impact of different devices for endovascular abdominal aortic aneurysm repair and related etiopathogenetic implications. Int Angiol. (2020) 39(5):398–404. 10.23736/S0392-9590.20.04163-2 32401471

[21] Steinmetz LM Coselli JS. Endovascular repair in patients with Marfan syndrome: concerns amid controversy. Ann Vasc Surg. (2023) 94:1–7. 10.1016/j.avsg.2022.04.049 35595210

[22] Yagyu T Noguchi T. Diagnosis and treatment of cardiovascular disease in patients with heritable connective tissue disorders or heritable thoracic aortic diseases. Cardiovasc Interv Ther. (2024) 39(2):126–36. 10.1007/s12928-023-00977-0 38182694

[23] Coselli JS Green SY Price MD Hash JA Ouyang Y Volguina IV Results of open surgical repair in patients with Marfan syndrome and distal aortic dissection. Ann Thorac Surg. (2016) 101(6):2193–201. 10.1016/j.athoracsur.2015.11.008 26876340

[24] Omura A Tanaka A Miyahara S Sakamoto T Nomura Y Inoue T Early and late results of graft replacement for dissecting aneurysm of thoracoabdominal aorta in patients with Marfan syndrome. Ann Thorac Surg. (2012) 94(3):759–65. 10.1016/j.athoracsur.2012.04.061 22818967

[25] Cuozzo S Marzano A Martinelli O Jabbour J Molinari A Brizzi V Early experience with inner branch stent-graft system for endovascular repair of thoraco-abdominal and pararenal abdominal aortic aneurysm. Diagnostics (Basel). (2024) 14(23):2612. 10.3390/diagnostics14232612 39682521 PMC11640084

[26] Irace L Laurito A Venosi S Irace FG Malay A Gossetti B Mid- and long-term results of endovascular treatment in thoracic aorta blunt trauma. ScientificWorldJournal. (2012) 2012:396873. 10.1100/2012/396873 22645421 PMC3356706

[27] Amako M Spear R Clough RE Hertault A Azzaoui R Martin-Gonzalez T Total endovascular aortic repair in a patient with Marfan syndrome. Ann Vasc Surg. (2017) 39:289.e9–289.e12. 10.1016/j.avsg.2016.07.069 27890843

[28] Kölbel T Eleshra A Aldag M Rohlffs F Debus SE Honig S Endovascular treatment of aortic pathologies in patients with Marfan syndrome: single-center experience. J Endovasc Ther. (2022) 29(4):602–10. 10.1177/15266028211067733 34969304

[29] Kouchoukos NT. Endovascular surgery in Marfan syndrome: cON. Ann Cardiothorac Surg. (2017) 6(6):677–81. 10.21037/acs.2017.10.05 29270380 PMC5721107

[30] Harky A Hussain SMA MacCarthy-Ofosu B Ahmad MU. The role of thoracic endovascular aortic repair (TEVAR) of thoracic aortic diseases in patients with connective tissue disorders - A literature review. Braz J Cardiovasc Surg. (2020) 35(6):977–85. 10.21470/1678-9741-2019-0367 33306324 PMC7731863

[31] Pacini D Parolari A Berretta P Di Bartolomeo R Alamanni F Bavaria J. Endovascular treatment for type B dissection in Marfan syndrome: is it worthwhile? Ann Thorac Surg. (2013) 95(2):737–49. 10.1016/j.athoracsur.2012.09.059 23273625

[32] Glebova NO Cameron DE. Black JH 3rd. Treatment of thoracoabdominal aortic disease in patients with connective tissue disorders. J Vasc Surg. (2018) 68(4):1257–67. 10.1016/j.jvs.2018.06.199 30244929

[33] Preventza O Mohammed S Cheong BY Gonzalez L Ouzounian M Livesay JJ Endovascular therapy in patients with genetically triggered thoracic aortic disease: applications and short- and mid-term outcomes. Eur J Cardiothorac Surg. (2014) 46(2):248–53; discussion 253. 10.1093/ejcts/ezt636 24477738

[34] Olsson KW Mani K Burdess A Patterson S Scali ST Kölbel T Outcomes after endovascular aortic intervention in patients with connective tissue disease. JAMA Surg. (2023) 158(8):832–9. 10.1001/jamasurg.2023.2128 37314760 PMC10267845

[35] Conway AM Qato K Anand G Mondry L Giangola G Carroccio A. Endovascular abdominal aortic aneurysm repair in patients with Marfan syndrome. Vascular. (2020) 28(1):48–52. 10.1177/1708538119858045 31260381

[36] Svensson LG Kouchoukos NT Miller DC Bavaria JE Coselli JS Curi MA Expert consensus document on the treatment of descending thoracic aortic disease using endovascular stent-grafts. Ann Thorac Surg. (2008) 85(1 Suppl):S1–41. 10.1016/j.athoracsur.2007.10.099 18083364

[37] Grabenwöger M Alfonso F Bachet J Bonser R Czerny M Eggebrecht H Thoracic endovascular aortic repair (TEVAR) for the treatment of aortic diseases: a position statement from the European association for cardio-thoracic surgery (EACTS) and the European Society of Cardiology (ESC), in collaboration with the European association of percutaneous cardiovascular interventions (EAPCI). Eur J Cardiothorac Surg. (2012) 42(1):17–24. 10.1093/ejcts/ezs107 22561652

[38] Isselbacher EM Preventza O Hamilton Black J 3rd Augoustides JG Beck AW Bolen MA 2022 ACC/AHA guideline for the diagnosis and management of aortic disease: a report of the American Heart Association/American College of Cardiology joint committee on clinical practice guidelines. Circulation. (2022) 146(24):e334–482. 10.1161/CIR.0000000000001106 36322642 PMC9876736

[39] Wanhainen A Van Herzeele I Bastos Goncalves F Bellmunt Montoya S Berard X Boyle JR Editor’s choice – European society for vascular surgery (ESVS) 2024 clinical practice guidelines on the management of abdominal aorto-iliac artery aneurysms. Eur J Vasc Endovasc Surg. (2024) 67(2):192–331. 10.1016/j.ejvs.2023.11.002 38307694

[40] Aru RG Richie CD Badia DJ Romesberg AM Sheppard MB Minion DJ Hybrid repair of type B aortic dissection with thoracoabdominal aortic aneurysmal degeneration in the setting of Marfan syndrome. Vasc Endovascular Surg. (2021) 55(6):619–22. 10.1177/1538574420988279 33627054

[41] Pellenc Q Girault A Roussel A De Blic R Cerceau P Raffoul R Optimising aortic endovascular repair in patients with Marfan syndrome. Eur J Vasc Endovasc Surg. (2020) 59(4):577–85. 10.1016/j.ejvs.2019.09.501 31865029

[42] Han JY James HI 3rd Manesh M Pyun AJ Miranda E Han SM. Hybrid approach to achieve secure distal seal zones during endovascular aortic repair in a patient with Marfan syndrome. J Vasc Surg Cases Innov Tech. (2024) 10(6):101595. 10.1016/j.jvscit.2024.101595 39282211 PMC11401353

[43] Fazzini S Torsello G Austermann M Beropoulis E Munaò R Torsello GF. Aortic endograft and bridging stent-graft remodeling after branched endovascular aortic repair. Vascular. (2021) 29(6):808–16. 10.1177/1708538120983698 33375927

[44] Squizzato F Oderich GS Bower TC Mendes BC Kalra M Shuja F Long-term fate of aortic branches in patients with aortic dissection. J Vasc Surg. (2021) 74(2):537–546.e2. 10.1016/j.jvs.2021.01.055 33592297 PMC8316268

[45] de Niet A Post RB Reijnen MMPJ Zeebregts CJ Tielliu IFJ. Geometric changes over time in bridging stents after branched and fenestrated endovascular repair for thoracoabdominal aneurysm. J Vasc Surg. (2019) 70(3):702–9. 10.1016/j.jvs.2018.12.023 30837180

[46] Ágg B Szilveszter B Daradics N Benke K Stengl R Kolossváry M Increased visceral arterial tortuosity in Marfan syndrome. Orphanet J Rare Dis. (2020) 15(1):91. 10.1186/s13023-020-01369-w 32293489 PMC7160945

[47] Hagerty T Geraghty P Braverman AC. Abdominal aortic aneurysm in Marfan syndrome. Ann Vasc Surg. (2017) 40:294.e1–e6. 10.1016/j.avsg.2016.07.067 27894713

[48] Kang J Kim YW Kim DK Woo SY Park YJ. Comparable surgical outcomes of abdominal aortic aneurysm repair in patients with and without Marfan syndrome. J Vasc Surg. (2021) 74(4):1163–71. 10.1016/j.jvs.2021.03.040 33887426

[49] Pavasini R Sanguettoli F Deserio MA Bianchi N Zanarelli L Fabbri G Drug-based cardiovascular prevention in patients with Marfan syndrome: a systematic review. Minerva Cardiol Angiol. (2023) 71(6):611–21. 10.23736/S2724-5683.23.06184-7 36939732

[50] Marcos-Ríos D Rochano-Ortiz A San Sebastián-Jaraba I Fernández-Gómez MJ Méndez-Barbero N Oller J. Mitochondrial dysfunction: a new hallmark in hereditable thoracic aortic aneurysm development. Cells. (2025) 14(8). 10.3390/cells14080618 PMC12026022 40277943

[51] Oller J Gabandé-Rodríguez E Ruiz-Rodríguez MJ Desdín-Micó G Aranda JF Rodrigues-Diez R Extracellular tuning of mitochondrial respiration leads to aortic aneurysm. Circulation. (2021) 143(21):2091–109. 10.1161/CIRCULATIONAHA.120.051171 33709773 PMC8140666

[52] van Andel MM Bosshardt D Schrauben EM Merton R van Kimmenade RRL Scholte A Effects of resveratrol on aortic growth in patients with Marfan syndrome: a single-arm open-label multicentre trial. Heart. (2024) 111(1):11–7. 10.1136/heartjnl-2024-324343 39317438 PMC11671954

[53] Rodríguez-Rovira I Arce C De Rycke K Pérez B Carretero A Arbonés M Allopurinol blocks aortic aneurysm in a mouse model of Marfan syndrome via reducing aortic oxidative stress. Free Radic Biol Med. (2022) 193(Pt 2):538–50. 10.1016/j.freeradbiomed.2022.11.001 36347404

